# Letter from Dr. Harvey

**Published:** 1860-01

**Authors:** C. H. Harvey


### ARTICLE VIII.

### *Letter from Dr. Harvey.*

BUFFALO, Nov. 14th, 1859.

DR. HARRIS:

*Dear Sir*—An article published in the November number, of the Dental Cosmos, on the subject of arresting excessive hemorrhage, sometimes following the extraction of teeth, has stimulated me to communicate to you for publication, should you deem it of sufficient interest to your readers, the following method of arresting the kind of hemorrhage spoken of.

Some twenty years ago, by accident, I hit or blundered upon a proccss that can never fail, if properly performed, no matter how profusely the blood may flow. I have no doubt you will think that I have been derelict in duty, in not publishing this before. The charge is acceded to, and the only reason, be that good or bad, that can be given, is a lazy constitutional repugnance, in writing upon any subject. The material used in plugging the socket or sockets, is simply lint, made from old or new black silk cloth. The silk being placed upon the knee, is scraped with a case knife, until a sufficient quantity is obtained.

Nut galls, alum and sulphate of iron, are the articles used in dying black silk. This lint, so strongly impregnated with these powerful astringents, forms with the blood, in its passage into the socket, a sure and certain compound, for a mechanical stoppage of the hemorrhage. The mode of introducing the lint is very simple. Take a sufficient quantity to fill the socket or sockets, up nearly even with the margin of the gum, lay the lint over the sockets you intend to plug. Take a good strong plugger, that will enable you to carry the lint down to within an eighth, or quarter of an inch of the apex of the socket. Introduce the lint continously, and in quite large quantities. Pack with force enough to make it quite solid. Now let the patient rinse the mouth, that you may ascertain if the bleeding is arrested. If not, use more pressure, and it may be necessary to force more lint by the side of that already there. This plug must be allowed to come away of itself, which will be in five or six days. More reliance is placed upon obtaining a firm plug than upon the astringent qualities of the silk. The dyes of the silk seem to render it capable of adhering together, and of remaining in its place.

Yours, truly,

C. H. HARVEY.

